# The pathogenic mechanism of syndactyly type V identified in a *Hoxd13*Q50R knock-in mice

**DOI:** 10.1038/s41413-024-00322-y

**Published:** 2024-04-01

**Authors:** Han Wang, Xiumin Chen, Xiaolu Meng, Yixuan Cao, Shirui Han, Keqiang Liu, Ximeng Zhao, Xiuli Zhao, Xue Zhang

**Affiliations:** 1 McKusick-Zhang Center for Genetic Medicine, State Key Laboratory of Complex Severe and Rare Diseases, Department of Medical Genetics, Institute of Basic Medical Sciences Chinese Academy of Medical Sciences, School of Basic Medicine Peking Union Medical College, Beijing, 100005 China; 2 Department of Orthopedics, State Key Laboratory of Complex Severe and Rare Diseases, Peking Union Medical College Hospital, Chinese Academy of Medical Sciences & Peking Union Medical College, Beijing, 100730 China

**Keywords:** Bone, Pathogenesis

## Abstract

Syndactyly type V (SDTY5) is an autosomal dominant extremity malformation characterized by fusion of the fourth and fifth metacarpals. In the previous publication, we first identified a heterozygous missense mutation Q50R in homeobox domain (HD) of *HOXD13* in a large Chinese family with SDTY5. In order to substantiate the pathogenicity of the variant and elucidate the underlying pathogenic mechanism causing limb malformation, transcription-activator-like effector nucleases (TALEN) was employed to generate a *Hoxd13*Q50R mutant mouse. The mutant mice exhibited obvious limb malformations including slight brachydactyly and partial syndactyly between digits 2–4 in the heterozygotes, and severe syndactyly, brachydactyly and polydactyly in homozygotes. Focusing on BMP2 and SHH/GREM1/AER-FGF epithelial mesenchymal (e-m) feedback, a crucial signal pathway for limb development, we found the ectopically expressed *Shh*, *Grem1* and *Fgf*8 and down-regulated *Bmp2* in the embryonic limb bud at E10.5 to E12.5. A transcriptome sequencing analysis was conducted on limb buds (LBs) at E11.5, revealing 31 genes that exhibited notable disparities in mRNA level between the *Hoxd13*Q50R homozygotes and the wild-type. These genes are known to be involved in various processes such as limb development, cell proliferation, migration, and apoptosis. Our findings indicate that the ectopic expression of *Shh* and *Fgf8*, in conjunction with the down-regulation of *Bmp2*, results in a failure of patterning along both the anterior-posterior and proximal-distal axes, as well as a decrease in interdigital programmed cell death (PCD). This cascade ultimately leads to the development of syndactyly and brachydactyly in heterozygous mice, and severe limb malformations in homozygous mice. These findings suggest that abnormal expression of *SHH, FGF8*, and *BMP2* induced by *HOXD13*Q50R may be responsible for the manifestation of human SDTY5.

## Introduction

Syndactyly is one of the most common hereditary limb malformations with significant genetic and phenotypic heterogeneity. To date, at least nine types of non-syndromic syndactyly have been described, and the causative genes for types II–1, III, IV, V, and VII have been identified. The hallmark of syndactyly type V (SDTY5; OMIM 186300) is the fusion of the fourth and fifth metacarpals.^1^ In previous study, we firstly found that *HOXD13* was the pathogenic gene responsible for SDTY5 by genetic study in a six-generation Chinese Han family, and confirmed that c.950A>G (p.Q317R) or Q50R in homeodomain (HD) in *HOXD13* was the pathogenic mutation of SDTY5.^2^ The *HOXD13* gene comprises two exons: exon 1 comprises a 45-bp trinucleotide repeat encoding a 15-residue polyalanine, and exon 2 encodes a highly conserved homeodomain.^3^ The polyalanine tract expansion in *HOXD13*, responsible for SDTY2 (OMIM 186000), was the first pathogenic mutation identified in *HOX* genes.^4^ An I47L substitution in the *HOXD13* homeodomain causes brachydactyly and central polydactyly by producing a selective loss of function, however the pathogenic mechanism of *HOXD13* variant Q50R leading to V-type syndactyly is not yet clear.^5^

Limb buds (LBs) patterning is organized along three axes, and each of these axes develops under the control of a specific signaling center. The proximal-distal (P-D) axis, extending from the body trunk to the tip of the digits, is controlled by the apical ectodermal ridge (AER, a structure formed at the distal intersection of the dorsal and ventral ectoderm). The anterior-posterior (A-P) axis, ranging from the thumb to the little finger, is regulated by the zone of polarizing activity (ZPA, a small group of mesenchyme cells at the posterior margin of the vertebrate limb bud). The dorsal-ventral (D-V) axis, from the dorsum to the palm of the hand, is patterned by non-AER ectoderm.^6^ *Shh* is a polarizing morphogen expressed in the ZPA and is required for limb growth and patterning.^7^ Studies have reported when posterior *Shh*-expressing cells are grafted to the anterior margin of chick wing buds, they induce mirror-image duplications of a second limb.^8^ *Shh* expression leads to the activation of *Hox* genes.^9^ *5’-Hox* gene expression also controls limb bud A-P polarity, thereby facilitating posterior-restricted expression of *Shh* to generate the ZPA and determine posterior LBs patterning.^10^ In the mesenchyme of posterior LBs, *Shh* expression is activated by binding of HOXD13 transcription factors to a distal *Shh* enhancer.^11^ Fibroblast growth factors (FGFs), mainly *Fgf4*, *Fgf8*, *Fgf9*, and *Fgf17*, display AER-specific expression domains within the LBs to direct outgrowth along the P-D axis.^12^ Among these four *Fgf* genes, *Fgf8* is the only gene expressed throughout the whole AER.^13^ Both *Shh* and *Fgf8* are required to activate *Hoxd13* in limb mesenchymal cells.^14^

Bone morphogenetic protein (BMP) signaling is essential for normal formation of the AER and plays a role in programmed cell death (PCD).^15^ A typical model of PCD during embryogenesis is a developing limb, in which PCD is necessary to remove interdigit tissue to allow proper separation of the digits to prevent the onset of syndactyly.^16^ In fact, the ZPA and AER signaling centers are linked to each other. *Shh* expressed in the ZPA causes the upregulation of the BMP inhibitor Gremlin (GREM1). The self-regulatory *SHH*/*GREM1*/AER-FGF feedback signaling system controls LB outgrowth and patterning. Initially, the 5′-HOXD transcription factor regulates SHH expression. SHH promotes the expression of GREM1—a repressor of BMP—and therefore inhibits the expression of BMPs in the interfinger (toe) zone. BMPs repress the expression of the FGFs, which in turn enhances SHH expression in the ZPA.^17^ *Grem1* overexpression specifically blocks chick phalanx development by inhibiting the phalanx forming region (PFR) activity, and downregulation of *Grem1* in the distal limb mesoderm is necessary for phalanx development.^18^

Transcription-activator-like effector nuclease (TALEN) refers to engineered, sequence-specific DNA-binding proteins (TALEs) that are bound to a nonspecific endonuclease domain (FokI), and are used as gene editing tools for site-specific induction of DNA double-strand breaks (DSBs).^19,20^ After specific programming of TALEs, they can be used for the recognition of a target-specific nucleotide.^21^ In order to further identify the pathogenicity of the mutation found in a SDTY5 family and to clarify the pathogenic mechanisms of SDTY5, we generated *Hoxd13*Q50R knock-in mutant mice by TALEN with the aim to reveal the underlying regulating network involving *HOXD13* in the development of SDTY5.

## Results

### The establishment of *Hoxd13*Q50R mutant mice by TALEN technique

**Fig. 1:**
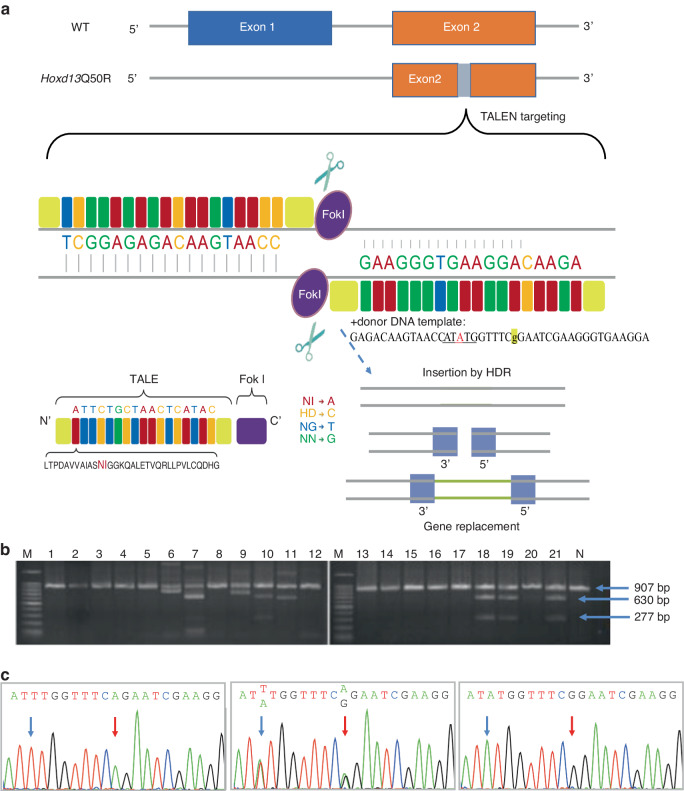
The establishment of *Hoxd13*Q50R knock-in mice. **a** Schematic illustration of mutation knock-in using the TALEN system. **b** No.10, 18, 19, and 21 of first-generation mice were confirmed to carry a heterozygous mutation of *Hoxd13*Q50R by *Nde*I digestion. (M = DNA marker, N = naïve mice). **c** Sanger-sequencing result of wild-type (WT/WT), *Hoxd13*Q50R heterozygous mice (Q50R/WT), and *Hoxd13*Q50R homozygous mice (Q50R/Q50R)

In order to achieve efficient genome editing of the murine ortholog of the human *HOXD13* gene, we designed a pair of TALEs targeting the second exon of the murine *Hoxd13* gene. Our objective was to induce an A-to-G mutation at position 1 769 (50 position in *Hoxd13* homeodomain). Additionally, to facilitate genotyping of the mice in subsequent experiments, we incorporated a single *Nde*I site into the donor DNA template by introducing a T-to-A mutation at the eighth nucleotide upstream of the target site (as depicted in Fig. 1a). It is worth noting that this introduced variant corresponds to a synonymous change, thereby encoding the same wild-type amino acid.

In total, we obtained 21 F0 generation mice and subsequently identified four individuals (designated as F0-10, F0-18, F0-19, and F0-21) harboring the *Hoxd13*Q50R mutation via single restriction enzyme *Nde*I digestion (Fig. 1b). Subsequently, PCR-Sanger sequencing was used to identify potential off-target effects in exon2 of genes(*Hoxa2*, *Hoxa10*, *Hoxa11*, *Hoxb9*, *Hoxb13*, *Hoxc8*, *Hoxc11*, *Hoxd9*) exhibiting high homology with *Hoxd13*. Our results confirmed that the *Hoxd13*Q50R mutation was the sole mutation induced in the four identified F0 generation mutant mice. By mating *Hoxd13*Q50R heterozygous female and male mice, we obtained *Hoxd13*Q50R homozygous mice. Figure 1c shows the genotypes of the wild-type, heterozygote, and homozygote.

### *Hoxd13*Q50R mutant mice display limb malformations

**Fig. 2:**
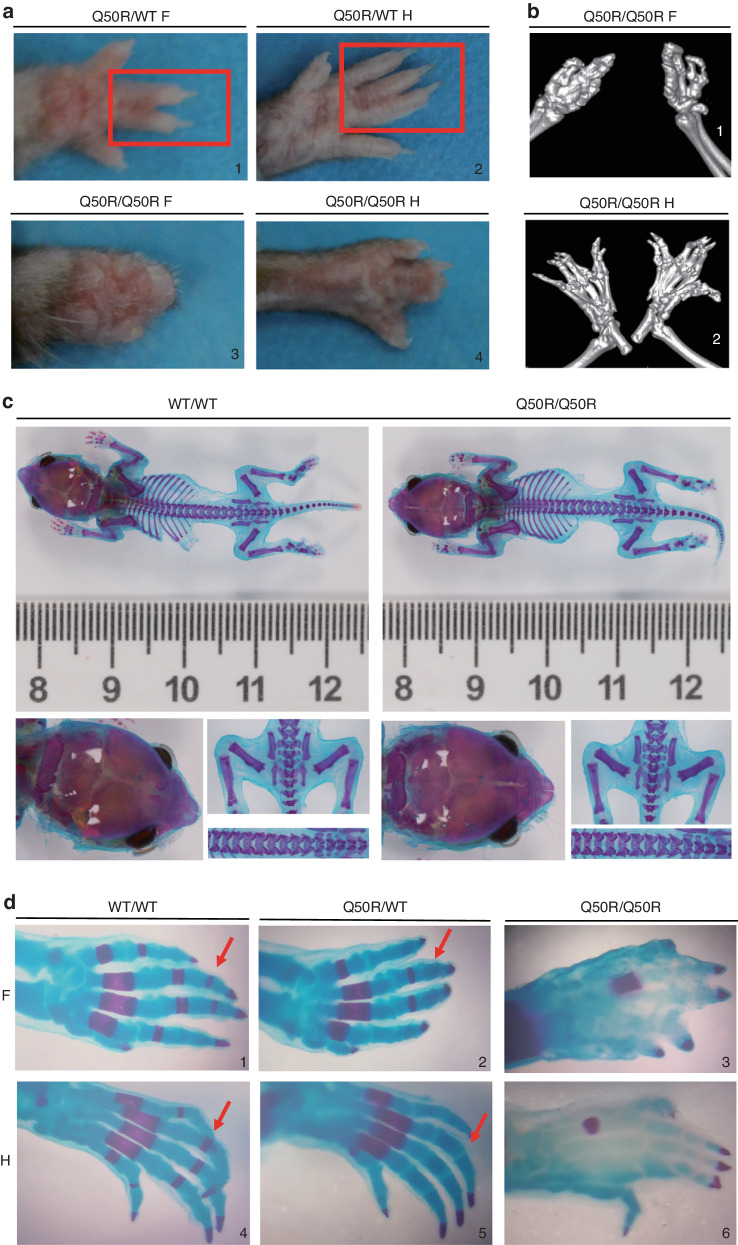
Morphological analyses of the mice with different genotypes. **a** Macroscopic results. 2a1 and 2a2 show cutaneous or bony syndactyly between digits 2–4 in Q50R/WT mice forelimb (F) and hindlimb (H), respectively. 2a3 and 2a4 show truncated forelimb and central syndactyly hindlimb in Q50R/Q50R mice (red boxes marked syndactyly in Q50R/WT). **b** Micro CT scanning results. 2b1 show syndactyly with webbing and no distinct phalanges in Q50R/Q50R mice forelimb. 2b2 show central syndactyly and brachydactyly in the hindlimb of Q50R/Q50R mice. **c** Skeletal preparations of WT/WT and Q50R/Q50R at postnatal day 4(P4). **d** Skeletal preparations of WT/WT, Q50R/WT, and Q50R/Q50R at postnatal day 0(P0) (red arrows marked different ossification levels of phalanges between WT/WT and Q50R/WT). 2d1, 2d2, 2d4, and 2d5 show the delayed ossification of phalanges in the mice of Q50R/WT compared with the wild-type. 2d3 and 2d6 show the dysplasia and disorganization of bone and cartilage in Q50R/Q50R’s autopods

The limb of the mouse consists of five digits that follow an antero-posterior sequence (denoted as 1, 2, 3, 4, and 5). Of these, digits 2-5 exhibit a high degree of morphological similarity due to each possessing three phalanges.^8^ Notably, we have observed a mild manifestation of brachydactyly and syndactyly, either cutaneous or bony, between digits 2 and 4 in adult heterozygous mice with *Hoxd13*Q50R mutation (Fig. 2a1, a2). As shown in Table 1, we performed a statistical analysis of the proportion of different degrees of syndactyly in 32 heterozygous *Hoxd13*Q50R mice. All homozygous mice of *Hoxd13*Q50R display a more severe phenotype characterized by syndactyly with webbing and lack of distinct phalanges in the forelimbs, and central syndactyly and brachydactyly in the hindlimbs (Fig. 2a3, a4, and b1). Micro computed X-ray tomography(micro CT) scanning revealed the presence of six digits in the hindlimbs of the homozygous mice (Fig. 2b2).

**Table 1:** Distribution of syndactyly manifestation in 32 heterozygous mice with *Hoxd13*Q50R

Forelimb		Hindlimb		Count (ratio)
Right	Left	Right	Left	Total: 32(100%)
NA	NA	Digits 3,4	NA	2(6.25%)
NA	NA	Digits 2, 3, 4	NA	1(3.125%)
NA	NA	Digits 2–4	NA	1(3.125%)
NA	NA	Digits 2–4	Digits 2, 3	7(21.875%)
NA	NA	Digits 2–4	NA	1(3.125%)
Digits 2, 3	NA	Digits 2–4	NA	1(3.125%)
Digits 2, 3	Digits 2, 3	Digits 2–4	NA	9(28.125%)
Digits 2, 3	Digits 2, 3	NA	NA	1(3.125%)
NA	NA	NA	NA	9(28.125%)

The animals at postnatal day 4 (P4) were subjected to skeletal preparations and subsequently analyzed. No significant differences were found in the development and structure of long bones, and cranial bones between wild-type mice and homozygous mice. However, statistical analysis revealed that the vertebral column length of homozygous mice was 1–4 mm shorter than that of wild-type mice, with a significance level of *P* < 0.001. (Fig. 2c, Table S1 and Fig. S1). Furthermore, the comparison between *Hoxd13*Q50R heterozygotes and their wild-type counterparts showed a delayed ossification of phalanges in the former (Fig. 2d1, d2, d4, and d5). In contrast, the autopod of *Hoxd13*Q50R homozygotes exhibited dysplasia and disorganization of bone and cartilage (Fig. 2d3, d6).

### Decreased BMP signaling regulates interdigit PCD, leading to syndactyly in *Hoxd13*Q50R

Firstly, we aimed to elucidate the pathogenic correlation between SDTY5 and the *Hoxd13*Q50R mutation during embryonic development. In a developing limb, critical regions of programmed cell death (PCD) are situated in the ectoderm of the AER and the undifferentiated mesoderm, concomitant with the establishment of prechondrogenic condensation of the skeleton.^22^ The bone morphogenetic protein (BMP) signaling pathway plays a positive role in regulating interdigital PCD.^23^

**Fig. 3:**
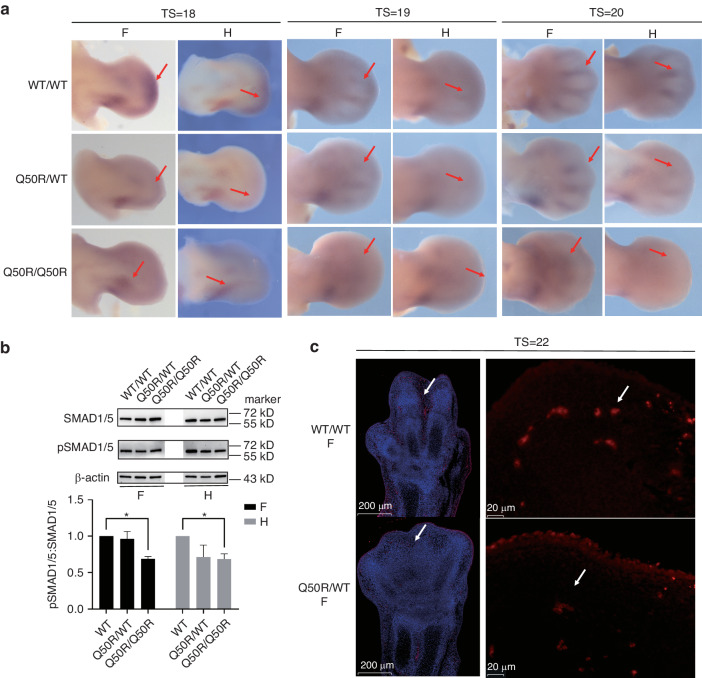
Gene expression analysis in BMP signaling. **a** The expression levels of *Bmp2* during various developmental stages of limb buds. The experimental groups include the mice of wild-type (WT), heterozygotes (Q50R/WT), and homozygotes (Q50R/Q50R). The forelimbs and hindlimbs are represented by “F” and “H,” respectively. “TS” denotes the time with somite numbers. The signals of *Bmp2* expression were detected by WISH indicated by red arrows. **b** Western blot analysis using SMAD1/5 and Phospho-SMAD1/5 in forelimbs (F) and hindlimbs (H) of WT/WT, Q50R/WT and Q50R/Q50R mice at E11.5. **c** TUNEL staining in interdigital regions of forelimbs from WT/WT and Q50R/Q50R mice at E13.5(TS = 22). Apoptotic cells were red. DAPI was used as a counterstain

To explore the potential involvement of BMP signaling in syndactyly development in *Hoxd13*Q50R mutant mice, we conducted an analysis of *Bmp2* mRNA expression. Specifically, we utilized whole-mount in situ hybridization (WISH) to visualize and quantify *Bmp2* expression patterns across several developmental stages of the mutant mice. The results indicated a clear down regulation of *Bmp2* expression in conjunction with ectopic expression in affected tissues (Fig. 3). At E11.0 (TS = 18), the LBs of wild-type exhibited posterior and distal expression of *Bmp2*, whereas the mutant mice showed only minor posterior expression. At E11.5 (TS = 19), *Bmp2* expression was observed between digits in the forelimbs and hindlimbs of both wild-type and Q50R/WT mice, but was not observed in Q50R/Q50R mice. The expression level of *Bmp2* in the distal regions of Q50R/WT and Q50R/Q50R mice was found to be gradiently decreased compared to the wild-type. At E12.0(TS = 20), *Bmp2* was expressed in posterior and interdigital regions, with downregulation of *Bmp2* expression in *Hoxd13*Q50R mutant mice, particularly in interdigital regions. In Q50R/Q50R mice, *Bmp2* expression in interdigital regions was barely detectable. *Bmp4* expression pattern was also examined at E11.5, but no transcriptional changes were observed (data not provided).

The critical event in BMP signal transduction involves the phosphorylation of transcription factors Smad1, 5, and 8 (referred to collectively as Smad1/5/8) through BMP receptor mediation, ultimately resulting in the transcription activation of BMP-induced gene in the nucleus.^24^ Total SMAD1/5 and phospho-SMAD1/5 were examined in hindlimbs of WT/WT, Q50R/WT and Q50R/Q50R mice at E11.5 by western blot and the ratio of pSMAD1/5:total SMAD1/5 in the limb buds of Q50R/Q50R was significant decreased than that in the wild-type (Fig. 3b). These data demonstrated that the decreased expression of *Bmp2* in the limbs of the mutant mice regulates the subsequent BMP signaling pathway by influencing the phosphorylation levels of SMAD1/5.

Subsequently, we employed the TUNEL staining method to detect apoptotic cells in the interdigital regions of E13.5 wild-type and homozygous mice. The signal intensity of apoptotic cells in the interdigital regions of wild-type mice was significantly stronger than that in the homozygous mice (Fig. 3c). This further elucidates that the downregulation of BMP signaling indeed affects the PCD in the interdigital regions, thereby resulting in syndactyly.

Based on these findings, it can be inferred that the reduced expression of *Bmp2* in the distal regions of *Hoxd13*Q50R mutant mice during the onset of limb patterning results in the untimely and irregular PCD of the interdigital tissue. This, in turn, interferes with the usual process of chondrogenesis and digit differentiation, ultimately resulting in anomalies such as syndactyly and limb malforamtions.

### The expression of the SHH/GREM1/AER-FGF epithelial-mesenchymal feedback signaling in *Hoxd13* mutant mice

**Fig. 4:**
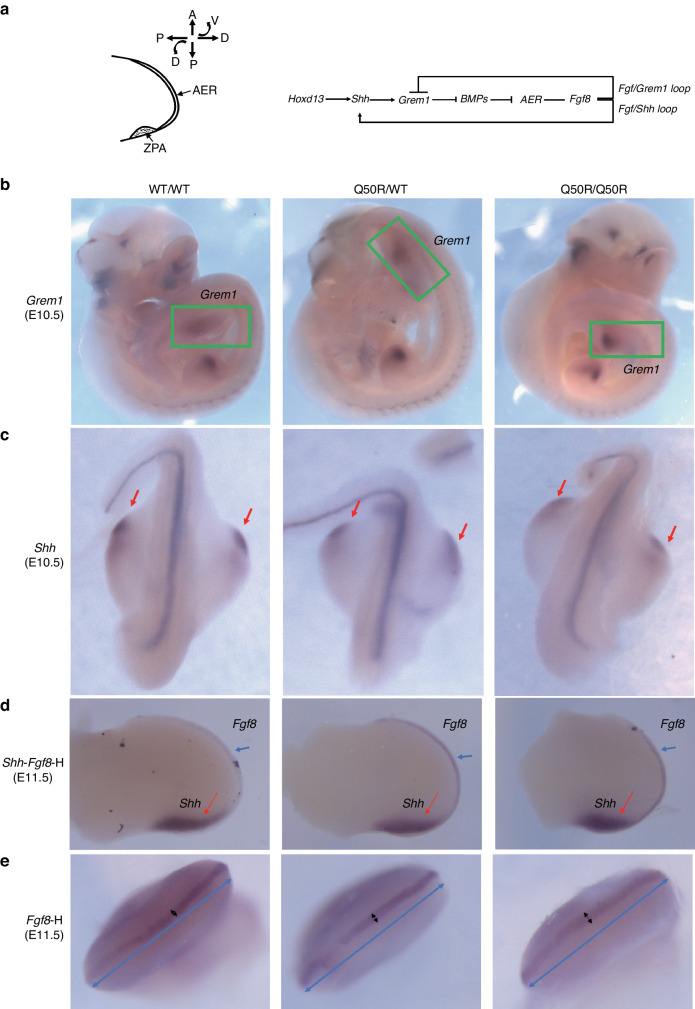
Gene expression analyses of SHH/GREM1/AER-FGF e-m signaling in limb buds by WISH. **a** The illustration of apical ectodermal ridge (AER) and zone of polarizing activity (ZPA) with A-P axis, P-D axis and D-V axis and the SHH/GREM1/AER-FGF e-m signaling feedback loop. **b** *Grem1* expression pattern in WT/WT, Q50R/WT, and Q50R/Q50R at E10.5 (green boxes marked *Grem1* signal). **c** *Shh* expression pattern in WT/WT, Q50R/WT, and Q50R/Q50R at E10.5 (red arrows marked *Shh* signal). **d** *Shh*-*Fgf8* expression pattern in WT/WT, Q50R/WT, and Q50R/Q50R at E11.5 (red arrows marked *Shh* signal; blue arrows indicated *Fgf8* signal). **e** *Fgf8* expression pattern in WT/WT, Q50R/WT, and Q50R/Q50R at E11.5 (black arrows marked *Fgf8* signal; blue double arrows were used as scale bars to indicate that the hindlimbs of WT/WT, Q50R/WT, and Q50R/Q50R were of the same size)

As previously mentioned, the A-P axis is formed through the activity of ZPA signaling centers that are under the control of the morphogenetic hormone SHH. The regulation of distal LB growth occurs through the SHH/GREM1/AER-FGF epithelial-mesenchymal (e-m) feedback signaling system^17^ (Fig. 4a). Within this feedback signaling system, the 5′-HOXD transcription factor initially regulates SHH expression, which subsequently promotes the expression of GREM1. GREM1 then represses the expression of BMPs in the interphalangeal region, acting as a BMP repressor.^22^ In Q50R/Q50R mice, limb patterning along both the A-P and P-D axes fails completely. Therefore, experiments were conducted to identify the expression patterns of the genes involved in the SHH/GREM1/AER-FGF e-m feedback signaling system.

Based on the previous analysis of *Bmp2* expression, we performed WISH to detect the expression pattern of BMP antagonist *Grem1* at the RNA level, and we did find a significant difference between Q50R/Q50R and wild-type mice at E10.5. The expression level of *Grem1* in Q50R/Q50R mice forelimbs was higher than that in wild-type (Fig. 4b). However, no obvious expression changes was detected at later development phases.

To evaluate the expression pattern of the *Grem1* upstream morphogen *Shh* and *Bmp2* downstream *Fgf8,* we performed a combined WISH analysis. Our findings revealed that *Shh* was strictly expressed in the zone of polarizing activity (ZPA) in wild-type mice. However, in *Hoxd13*Q50R mutant mice, we observed a slight diffusion of *Shh* expression sites around the ZPA at E10.5 (Fig. 4c). At E11.5, Q50R/Q50R mice had a shorter but broader *Shh* expression than WT/WT and Q50R/WT mice (Fig. 4d). Furthermore, our results indicated that *Fgf8* had a broader expression in the AER of *Hoxd13*Q50R mutant mice, which suggested that *Fgf8* was upregulated in the mutant mice (Fig. 4e) when compared to wild-type mice.

The ZPA is essential for developing the asymmetry in digits across the A-P axis, and *Shh* acts as a mediator of A-P patterning.^14,25^ *Fgf8* is one of the AER-*Fgfs* that is necessary for normal outgrowth and patterning of the limb.^13^ Early activated *HoxA;D* genes promote the maintenance of AER-FGFs by providing proximal identity to cells where they are expressed, and early activated *HoxA;D* genes ensure proper AER-FGF-dependent expression of late/distal *HoxA;D* genes.^26^ Our analysis indicates that the abnormal expression of *Shh* and *Fgf8*, combined with the downregulation of *Bmp2* in *Hoxd13* mutant mice, contributes to the failure of A-P and P-D axis patterning and a reduction in interdigital PCD. Ultimately, this leads to the development of the syndactyly and brachydactyly phenotype in Q50R/WT mice and severe limb malformations in Q50R/Q50R mice.

### RNA-sequencing analysis of LBs of mice at E11.5

Transcriptomic profiling was conducted on murine buds of forelimbs and hindlimbs from the mice of wild-type and *Hoxd13*Q50R homozygotes at E11.5. A total of three individual biological replicates were selected for both groups to ensure reproducibility and statistical significance.

**Fig. 5:**
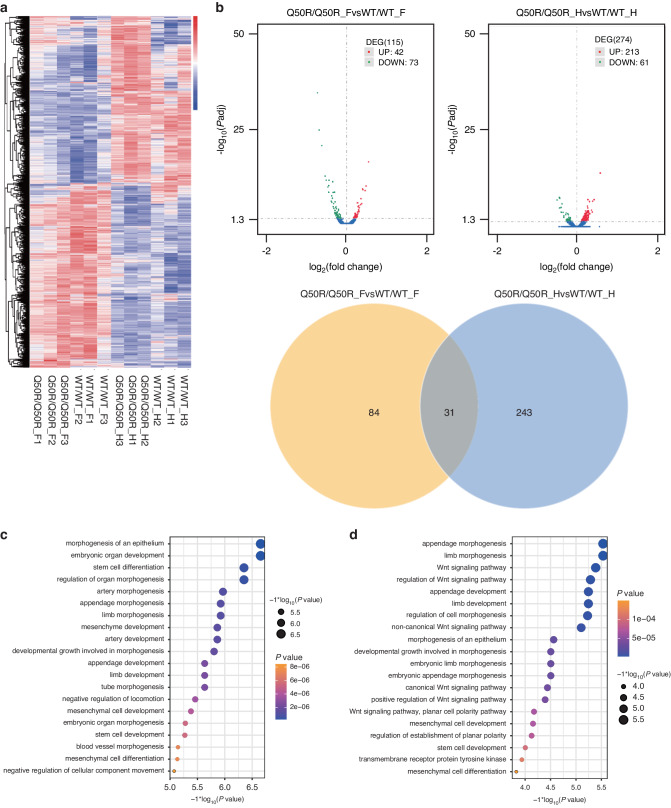
The results of RNA-sequencing analysis. **a** Clustering of DEGs in different groups. Red indicated highly expressed genes; blue indicated poorly expressed genes. **b** Volcano plot and venn diagram show the number of upregulated and downregulated genes in Q50R/Q50R_F vs WT/WT_F and Q50R/Q50R_H vs WT/WT_H, and their overlapping genes. DEGs were considered at *P*adj value < 0.05. **c** GO analysis of DEGs in the forelimbs of Q50R/Q50R and WT/WT mice. **d** GO analysis of DEGs in the hindlimbs of Q50R/Q50R and WT/WT mice

The RNA-sequencing analysis revealed significant differences in the expression of differentially expressed genes (DEGs) between the wild-type and homozygotes *of Hoxd13*Q50R (Fig. 5a). Based on the volcano plot analysis, there were 115 DEGs in the forelimbs between mice of Q50R/Q50R and wild-type, including 42 upregulated and 73 downregulated genes, and there were 274 DEGs in the hindlimbs between the mice of Q50R/Q50R and wild-type, with 213 upregulated genes, and 61 downregulated genes. 31 common DEGs were found, by combining the DEGs of the forelimbs and hindlimbs. According to *P*-values (*P* < 0.05), the 31 overlapped DEGs between the forelimbs and hindlimbs comprised *1190002N15Rik*, *Pcsk6*, *Plxna4*, *Tspan18*, *Lhx9*, *Cdc42ep3*, *Olfm1*, *Hoxd12*, *Evx2*, *Draxin*, *Smarcd2*, *Hey1*, *Tfap2b*, *Polg*, *Ppfibp2*, *Crmp1*, *Pam*, *Utrn*, *Bambi*, *Tbx4*, *Slc1a3*, *Cited2*, *Lhx2*, *Plod2, Klhl29*, *Slit3*, *Fzd8*, *Lix1*, *Pdgfrb*, *Sall1*, and *Scmh1* (Fig. 5b).

The GO functional enrichment analysis revealed that the DEGs in the forelimbs of Q50R/Q50R and WT/WT mice were primarily associated with various developmental processes such as epithelial cell morphogenesis, embryonic organ development, stem cell differentiation, regulation of organ morphogenesis, arterial morphogenesis, leg-tail morphogenesis, limb morphogenesis, mesenchymal development, arterial development, growth and development involved in morphogenesis, limb development, and vascular morphogenesis (Fig. 5c). The DEGs between Q50R/Q50R and WT/WT mice hindlimbs were mainly involved in processes such as limb morphogenesis, Wnt signaling pathway, regulation of cell morphogenesis, epithelial cell morphogenesis, mesenchymal cell development, transmembrane receptor protein tyrosine kinase signaling pathway, and embryonic limb morphogenesis (Fig. 5d).

**Fig. 6:**
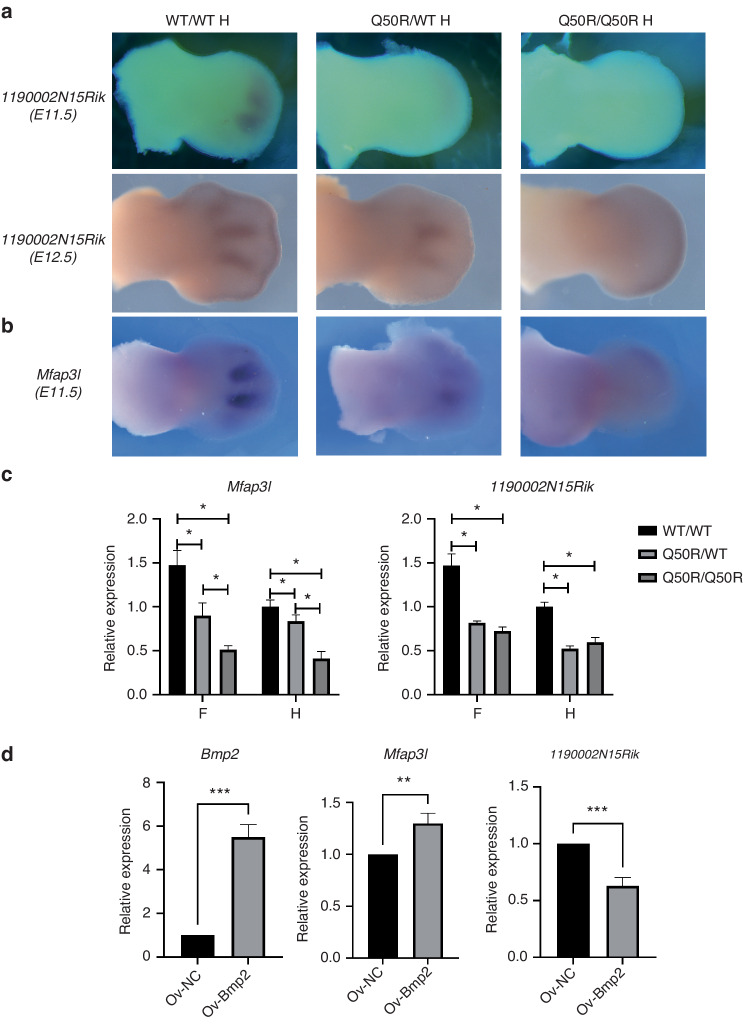
The expression of *1190002N15Rik* and *Mfap3l* in the LBs of mice with different genotypes. **a** The WISH results of *1190002N15Rik* expression pattern in WT/WT, Q50R/WT, and Q50R/Q50R hindlimbs at E11.5 and E12.5. **b** The WISH results of *Mfap3l* expression pattern in WT/WT, Q50R/WT, and Q50R/Q50R hindlimbs at E11.5. **c** RT-qPCR detection of the expression of *1190002N15Rik* and *Mfap3l* in forelimbs and hindlimbs of WT/WT, Q50R/WT, and Q50R/Q50R mice at E11.5. At least three replicates were performed. **d** RT-qPCR detection of the expression of *Mfap3l* and *1190002N15Rik* after overexpressing *Bmp2* in primary cultured chondrocytes from Q50R/Q50R mice (*P < 0.05)

Subsequently, a subset of common DEGs of forelimbs and hindlimbs between Q50R/Q50R and wild-type were validated using WISH and RT-qPCR. Specifically, *1190002N15Rik* encodes Golgi protein GoPro49, which is expressed in embryonic mesenchymal tissues. GoPro49 is known as a marker of the dental follicle and may have a function in the secretory pathway.^27^ In this study, we utilized WISH to investigate the expression pattern of *1190002N15Rik* and discovered that it exhibited initial expression in the distal 2–4 digit regions at E11.5, followed by strong expression between the metatarsals at E12.5. RT-qPCR analysis indicated a decreased expression level of *1190002N15Rik* in mutant mice compared to WT/WT, which suggests that mutant mice may display an abnormal rate of limb patterning (Fig. 6c). Furthermore, our observations revealed the absence of *1190002N15Rik* expression at E11.5, but we noted widespread ectopic expression in the distal regions of the limb at E12.5 in Q50R/Q50R mice (Fig. 6a). Microfibril-associated protein 3-like (MFAP3L), belonging to the MAGP family, is involved in regulating cell proliferation, migration, and invasion in cancer, as reported in previous studies.^28^ Our findings indicated a strong signal of *Mfap3l* in interdigit regions of the limbs in WT/WT, moderate expression in Q50R/WT, and a slight distal expression in Q50R/Q50R at E11.5 (Fig. 6b). Meanwhile, RT-qPCR results indicated that the expression levels of *Mfap3l* in the LBs of wild-type at E11.5 were significantly higher compared to *Hoxd13* mutant mice (Fig. 6c).

To evaluate the correlation between *1190002N15Rik*, *Mfap3l*, and *Bmp2*, we introduced a *Bmp2* overexpression vector into primary cultured chondrocytes derived from homozygous mutant mice. RT-qPCR analysis revealed a significant upregulation of *Mfap3l* expression and a concurrent downregulation of *1190002N15Rik* upon *Bmp2* overexpression (Fig. 6d). Conversely, overexpressing *MFAP3L* and *1190002N15Rik* in the human chondrocyte cell line C28/I2 did not induce significant alterations in *BMP2* expression compared to the control group (Fig. S2). These findings suggest that *Bmp2* may function as an upstream regulator of *Mfap3l* and *1190002N15Rik* during limb development. However, the precise regulatory associations between *Mfap3l* and *1190002N15Rik* and *Bmp2*, as well as *Hoxd13*, warrant further investigation and validation.

## Discussion

### *Hoxd13*Q50R mutant mice exhibit limb malformations

In mammals, *Hox* genes are highly conserved homeodomain transcription factors. They are distributed in *HOXA*, *HOXB*, *HOXC*, and *HOXD* clusters, and subdivided into 13 groups (from *HOX1* to *HOX13*) based on sequence homology and their positions in clusters.^29,30^ 5′-*HOXD* genes determine the correct formation of limb skeletal elements, and *HOXD13—*the most distal 5′-located *HOXD* gene—plays a decisive role in patterning the autopod (hand plate) region in early limb buds (LBs).^31^ Limb development depends on harmonious coordination between growth and patterning. 5′-*HOXD* genes and other related genes are involved in this complicated and highly choreographed process.^32^ In *Hoxa* and *Hoxd* knockout mouse models, limbs are arrested in early developmental patterning process and display severe truncation of distal element and absence of *Shh* expression.^33^

Several *Hoxd13* mutant animal models have been reported previously, such as spdh, a natural mutant mice model, which is caused by a polyalanine expansion in *Hoxd13* expresses severe delay in ossification and loss of joints; another model, a homozygous *hoxd13* knock-out mouse *Hoxd13*^–/–^ also exhibits a delay in ossification.^34^ Due to the highly conserved sequence of the *HOX* gene family, it is hard to establish an *Hoxd13* mice model containing a single point mutation in homeodomain by traditional gene targeting technology. In this study, TALEN was used to successfully develop the first *Hoxd13* mouse model with a point mutation Q50R—in homeobox domain.

*Hoxd13*Q50R mice displayed multiple limb malformations, which confirmed the relationship between *Hoxd13*Q50R and SDTY5. In our previous study, all SDTY5 affected were the heterozygotes of Q50R mutations in *HOXD13* homeodomain, and no homozygote was found of the same mutation. In another study, no obvious limb malformations were noted in the individuals with a heterozygous *HOXD13* missense mutation c.938C>G (p.T313R) or T46R in HD, but the homozygotes of T46R result in a severe SPD with metacarpal to carpal transformation.^35^ However, in our mouse model, all heterozygotes showed shortened digits, while some exhibited syndactyly and polydactyly, displaying different pheontypes campared to SDTY5 patients. The *Hoxd13*Q50R homozygous mice showed much more severe limb malformations than the heterozygotes, and the phenotypes were more similar to that of the human homozygotes of *HOXD13* HD-T46R. However, our heterozygous mice do not fully replicate the phenotypes of SDTY5 patients, only displaying slight brachydactyly and partial syndactyly which may be due to the different digit features and underlying different expressed genes involved in limb development between mice and humans. Furthermore, our statistical analysis revealed that hindlimb syndactyly was more common than forelimb syndactyly in *Hoxd13*Q50R heterozygous mice and all homozygous mice had webbed forelimbs and six digits in hindlimbs. The C57BL/6 mouse strain has four digits in the forelimb and five digits in the hindlimb, and the morphology of the digital bones also differs between the forelimb and hindlimb. In the development of mice, forelimbs initiate at E9.5, while hindlimbs start later and complete at E14.5.^36^ The development of hindlimbs generally lags behind forelimbs by about half a day. Additionally, the RNA-seq data of limb buds from mice at E11.5 showed that forelimbs and hindlimbs have different expression profiles. Due to these reasons, in mutant mouse model, there are phenotypic differences in the forelimbs and hindlimbs.

### The role of aberrant *Shh* and *Fgf8* expression and the reduced *Bmp2* function in SDTY5 development

In vertebrate limbs that lack webbing, the embryonic interdigit region is removed by PCD.^15^ BMP2/BMP4 protein induces apoptosis of mesenchymal cells isolated from the interdigital region in vitro. Inhibition of BMP signaling in chick embryos can result in excess web formation at the anterior and posterior regions of LBs in addition to marked suppression of the regression of webbing at the interdigital regions.^37^ Lower BMP production leads to digit loss as indeed observed in the BMP2&BMP4 conditional knock-out mice. A 30% increasing of BMP2 results in polydactyly and ectopically expressed BMP2 can induce duplication of digit 2 and bifurcation of digit 3.^38^ In this study, we detected downregulated *Bmp2* expression and lower phosphorylated Smad1/5 levels in distal regions of *Hoxd13*Q50R mutant mice during limb patterning. The subsequently delayed *Bmp2* interdigit expression blocked PCD in digit separation and eventually resulted in syndactyly and limb malformations.

The pattern and timing of *Hox* genes expression must be tightly controlled in animal development.^39^ In the SHH/GREM1/AER-FGF e-m feedback signaling system of limb development, HOXD13 is involved in this process as a transcription factor of SHH. We detected abnormal expression of *Shh* in ZPA in Q50R/Q50R mice at E11.5. *Shh* is required for the outgrowth of the limb. In mouse embryos lacking Shh function, only one digit-like structure develops in the hindlimbs, while no digits develop in the forelimbs.^8,40^ FGFs act as survival factors for the interdigit mesenchyme, and BMP signaling to the AER indirectly regulates interdigit PCD by regulating AER-FGFs. Increased Fgf expression by 50% results in supernumerary digits.^38^ We also found upregulated *Fgf8* expression in AER of the *Hoxd13*Q50R mutant mice limb buds, which was consistent with downregulated expression of *Bmp2*.

The RNA-Seq and WISH analyses revealed decreased expression of *1190002N15Rik* and *Mfap3l* in the process of limb patterning of *Hoxd13*Q50R mutant mice, while their precise function in limb development remains unknown.

In conclusion, we employed TALEN technology to successfully generate the initial *Hoxd13* mouse model with a Q50R point mutation located within the homeobox domain. Our study suggest that the limb malformations observed in the Chinese family with SDTY5 and *Hoxd13*Q50R mutant mice are the results of abnormal limb development. Our data support that the ectopic expression and down-regulation of *Bmp2* resulted in reduced phosphorylation levels of Smad1/5, leading to decreased apoptosis in the interdigital region and ultimately causing syndactyly malformations. Moreover, the aberrant expression of *Bmp2*, combined with abnormal expressions of *Shh* and *Fgf8*, further disrupts the spatiotemporal regulation network during limb bud outgrowth and patterning. Therefore, patients with SDTY5 and mice carrying the *Hoxd13*Q50R mutation exhibit complex limb malformations. In the transcriptome sequencing results, we identified some new genes such as *1190002N15Rik* and *Mfap3l* that may related to limb development. However, in this study, we did not extensively investigate these genes and their roles in limb development. Future research is warranted to elucidate their upstream and downstream interactions.

## Materials and methods

### Production of mutant embryos

The mice models were established by TALEN. The first 10 modules of TALEN1-F and TALEN1-R were ligated into FUS_A plasmids in the first step, and the remaining modules were ligated into FUS_B vectors, simplified as T1-FA, T1-RA, T1-FB, T1-RB. Bacteria liquid PCR and Sanger sequencing were conducted to select the right monoclonal colonies. Then, the correct FUS_A and FUS_B plasmids were subsequently joined into mammalian expression vector TAL-ELD and TAL-KKR. The ability to cleave the target site of the designed TALENs was evaluated by transient transfection. The TALEN mRNA was synthesized from plasmids linearized by *Xho*I and *Afl*II digestion and purified as previously described. After successfully constructing the TALEN gene editing system, 4–5-week-old female mice were induced by intraperitoneal injection of pregnant horse serum gonadotropin (PMSG) (10 IU) and human chorionic gonadotropin (HCG) 48 h after PMSG injection. Then, the female mice were transferred to cohabit with adult male mice overnight. In the next morning, the female mice with vaginal plugs were selected as donor mice to collect fertilized eggs. We injected the successfully constructed TALEN mRNA into the fertilized eggs’ cytoplasm, and transplanted the surviving fertilized eggs to pseudo-pregnant recipient mice’s fallopian tubes to generate F0 mutant mice.

To isolate embryos, timed pregnancies were set and the day of the vaginal plug was considered the gestational day zero. The somites of all isolated embryos were counted under stereomicroscope, and the embryonic stages were determined according to the counts of somites. For all experiments described in this study, age-matched embryos with an equal number of somites (variation: ± one somite) were used.

### Genotyping

DNA was extracted from embryonic membranes or tail tissue using the HotShot method for 15 min, and 2 μL DNA was used for PCR with LA Taq premix (TAKARA, Kusatsu, Japan). PCR conditions were as follows: 98 °C for 60 s followed by 35 cycles of 98 °C (10 s), 65 °C (10 s), and 72 °C (60 s). Primers for genotyping were as follows: *Hoxd13*-F2, ACAAATAACAAACGCACTC; *Hoxd13*-R2, GCGGTCCTAAATCACCTAATGC.

### Skeletal preparations

Newborn mice were sacrificed by air embolism. After peering anesthetic mice’s skin off by hot water, fixed them for 24 h in 95% ethanol. Then followed by overnight staining in 70% ethanol/5% acetic acid/0.015% Alcian Blue and 2–5 h in 95% ethanol after removing Alcian Blue. After treatment in 1% KOH, the samples were moved to 0.005% Alizarin Red/1% KOH for 48 h. Maceration was performed in 1% KOH/20% glycerol until mice were clear. After further clearing in 50% and 80% glycerol, the embryos were photographed on a Leica stereomicroscope.

### Micro-computed tomography

The adult mice were sacrificed by air embolism. The forelimbs and hindlimbs were scanned by microcomputed tomography (micro-CT; Siemens, Germany) to evaluate the difference between homozygous, heterozygous, and wild-type mice.

### Whole-mount in situ hybridization

Whole mouse embryos at E10.5-E12.5 were fixed in 4% paraformaldehyde overnight at 4 °C and stored in methanol at 20 °C until hybridization. WISH was performed using a modifier method from the protocol of Current Protocols in Molecular Biology (2004 edition).^41^ RNA probes were synthesized using vector-based probe synthesis: different genes’ fragment was amplified from cDNA of mice embryos at E11.5, using regular gene-specific primers. The PCR product was cloned into pGEM-T vector and linearized with *Not*I for antisense probe synthesis as described previously.^42^ All the probes were synthesized using this method. The probes uesd in this study were labeled with digoxin to detect the expression of *Bmp2*, *Bmp4*, *Shh*, *Fgf8*, *Grem1, 1190002N15Rik and Mfap3l*. Whole-mount images of embryos were taken using a Leica stereomicroscope.

### TUNEL staining

For tissue sections, the autopods were fixed in 4% PFA, washed in PBS and sectioned at 3–5 μm using a vibratome. BrdU-Red TUNEL Assay Kit (1:1 000, abcam, ab66110) to examine apoptosis in limb interdigits regions from WT/WT and Q50R/Q50R mice at E13.5. Subsequently, following the instructions provided by the assay kit, tissue slices from different samples were subjected to subsequent TUNEL staining procedures and imaged under a fluorescence microscope.

### Western blot

The E11.5 hindlimb buds of three genotypes were harvested for western blot analysis. The limbs were lysed in a denaturing lysis buffer containing protease inhibitors (Total Protein Extraction Kit, KeyGen Biotech, Nanjing, China) for 30 min on ice, and centrifuged (12 000 r/min) for 15 min at 4 °C. Lysate protein concentrations were determined using a BCA protein assay kit (Vazyme Biotech, Nanjing, China). Then, sample of 30 mg of protein was separated on a 12% sodium dodecyl sulfonate–polyacrylamide gel and transferred to a polyvinylidene fluoride membrane (Merck Millipore). The membrane was blocked using 5% nonfat dried milk in Tris-buffered saline with 0.1% Tween 20 (pH 7.6) for 1 h at RT, and incubated with pSMAD1/5 (1:1 000, Cell signaling, 9516S), SMAD1 (1:1 000, Cell signaling, 6944S), SMAD5 (1:1 000, Cell signaling, 9517) and β-Actin antibody (1:5 000, Bioworld, AP0060) overnight at 4 °C. The membrane was then incubated with horseradish peroxidase-conjugated secondary antibodies for 1 h at RT. Horseradish peroxidase was detected using an enhanced chemiluminescence detection system (ECL kit; Thermo Scientific, USA).

### Mesenchymal stem cells and chondrocytes differentiation

Mesenchymal stem cells (MSCs) and chondrocytes were derived from three-week C57BL/6 mice (at least three mice per group) femur. Briefly, bone marrow was flushed from femurs with PBS and then femur and cartilage were cut into small pieces to extrude cells with the use of 0.25% trypsin and 2 mg/mL collagenase II. The bone pieces were left undisturbed during 14 days until MSCs migrated to cell culture plate. MSCs and chondrocytes were cultured under standard growth medium (DMEM high glucose) with glutamax (Gibco, USA),100 μg/mL penicillin/streptomycin (Gibco), and fetal bovine serum at 10% (Gibco, USA).

### Over expression of *1190002N15Rik*, *MFAP3L* and *Bmp2*

Full-length cDNA of human *1190002N15Rik, MFAP3L* and mouse *Bmp2* was inserted into pmCherry-C1 vector, respectively. The plasmids were transformed into E.coli DH5α competent cells (TaKaRa, Shiga, Japan) and cultured in LB plates, and the sequences of these clones were verified by Sanger sequencing to ensure that there had no extra mutations. The clones with correct DNA sequence were cultured overnight in LB liquid medium, and plasmids were extracted using Endofree Maxi Plasmid Kit (TIANGEN, China). C28/I2 cells were cultured in complete Dulbecco’s Modified Eagle Medium (DMEM, Gibco) supplemented with 10% FBS and 1% penicillin-streptomycin, at 37 °C and 5% CO_2_. C28/I2 cells were seeded at a density of 5 × 10^5^ cells per well in 6-well cell culture plates before the day of transfection, and transfected with 1 μg DNA of *1190002N15Rik* and *MFAP3L* plasmids per well with lipofectamine™ 3000 reagents (Thermofisher, USA), following the protocol from manufacture. The *Bmp2* plasmid was transfected into primary cultured chondrocytes from Q50R/Q50R mutant mice.

### Quantitative RT-PCR

Total RNA was extracted and purified using Trizol reagent (Invitrogen, California, USA). A One Step SYBR® PrimeScript™ qPCR kit (TaKaRa Bio, Otsu, Japan) was used to synthesize cDNA following the manufacturer’s instructions. Quantitative real-time PCR (qPCR) was performed using SYBR® Premix Ex Taq™ (TaKaRa) in a Bio-Rad CFX96™ real-time PCR system using the following conditions: pre-denaturation at 95 °C for 5 s, followed by 40 cycles of denaturation at 95 °C, 10 s; annealing at 57 °C, 20 s; and extension at 72 °C, 20 s. The relative expression of the specified genes was calculated using the 2^−ΔΔCT^ method after normalization to *Gapdh* expression.

### RNA sequencing

The E11.5 limb buds obtained from the *Hoxd13*Q50R/Q50R and control mice were collected, and three specimens were prepared as the biological replicates of each genotype. Total RNA was isolated from the tissues by using TRIzol reagent (Ambion Life Technologies, Carlsbad, CA, USA). RNA-sequencing was performed on an Illumina Hiseq 2000/2500 platform (Chula Vista, CA, USA). Single-end clean reads were aligned to the reference genome by using Top Hat (v2.0.9, Johns Hopkins University, Baltimore, MD, USA). Between group differential gene expression was analyzed using the DEGseq (2010) R package (DESeq version 1.38.0. Eur. Mol. Biol. Lab. (2013)). Significantly different expression was defined as *P* < 0.05.

### Supplementary information

Supplemental table and figures
