# Supplementary material for: The pathogenic mechanism of syndactyly type V identified in a Hoxd13Q50R knock-in mice
Source: Bone Res. 2024 Apr 1;12:21. doi: 10.1038/s41413-024-00322-y (PMC10984994; doi:10.1038/s41413-024-00322-y)
Supplement: Supplementary file 1 — Supplemental table and figures [file 41413_2024_322_MOESM1_ESM.docx]

Supplemental table 1: Measurement of Body and Long Bone Lengths in WT/WT and Q50R/Q50R mice at P4 (measurement unit: mm)

| Sample NO. | Genotype | sex | Femur | Tibia/  Fibula | Radius/  ulna | Humerus | Cranial bone | Vertebral column |
| --- | --- | --- | --- | --- | --- | --- | --- | --- |
| 1 | Q50R/Q50R |  | 4 | 5 | 5 | 5 | 13 | 20 |
| 2 | Q50R/Q50R | ♂ | 4 | 5 | 5 | 5 | 12 | 20 |
| 3 | Q50R/Q50R | ♂ | 4 | 5 | 5 | 5 | 14 | 21 |
| 8 | Q50R/Q50R | ♀ | 4 | 6 | 5 | 5 | 14 | 22 |
| 9 | Q50R/Q50R | ♀ | 4 | 5 | 6 | 5 | 13 | 21 |
|  |  |  |  |  |  |  |  |  |
| 4 | Q50R/WT | ♂ | 4 | 5 | 5 | 5 | 14 | 24 |
| 10 | Q50R/WT | ♀ | 4 | 6 | 5 | 5 | 14 | 23 |
| 12 | Q50R/WT | ♀ | 4 | 5 | 5 | 5 | 14 | 24 |
| 13 | Q50R/WT | ♂ | 4 | 5 | 5 | 5 | 14 | 23 |
|  |  |  |  |  |  |  |  |  |
| 15 | WT/WT | ♂ | 4 | 5 | 5 | 4 | 14 | 24 |
| 16 | WT/WT | ♀ | 4 | 5 | 5 | 5 | 14 | 23 |
| 17 | WT/WT | ♂ | 4 | 5 | 5 | 5 | 13 | 24 |
| 19 | WT/WT | ♂ | 4 | 5 | 5 | 4 | 14 | 23 |

Supplemental Figs :


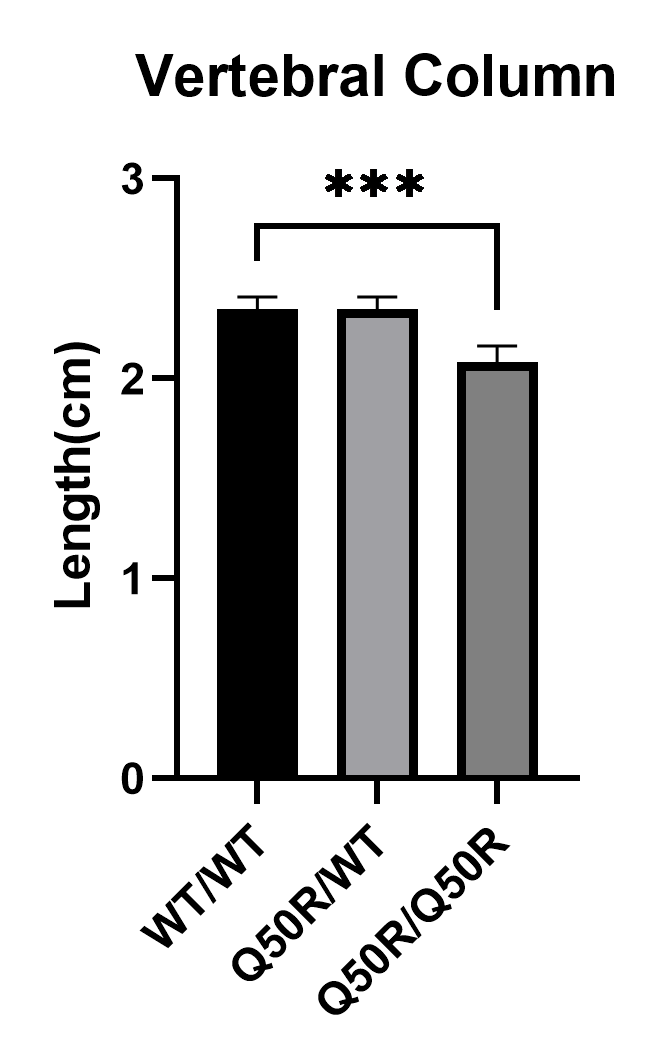


Fig S1 Statistical results of vertebral column lengths (including cervical vertebrae, thoracic vertebrae , lumbar vertebrae and sacral vertebrae) in the mice of wild type, mutant heterozygote, and mutant homozygote.


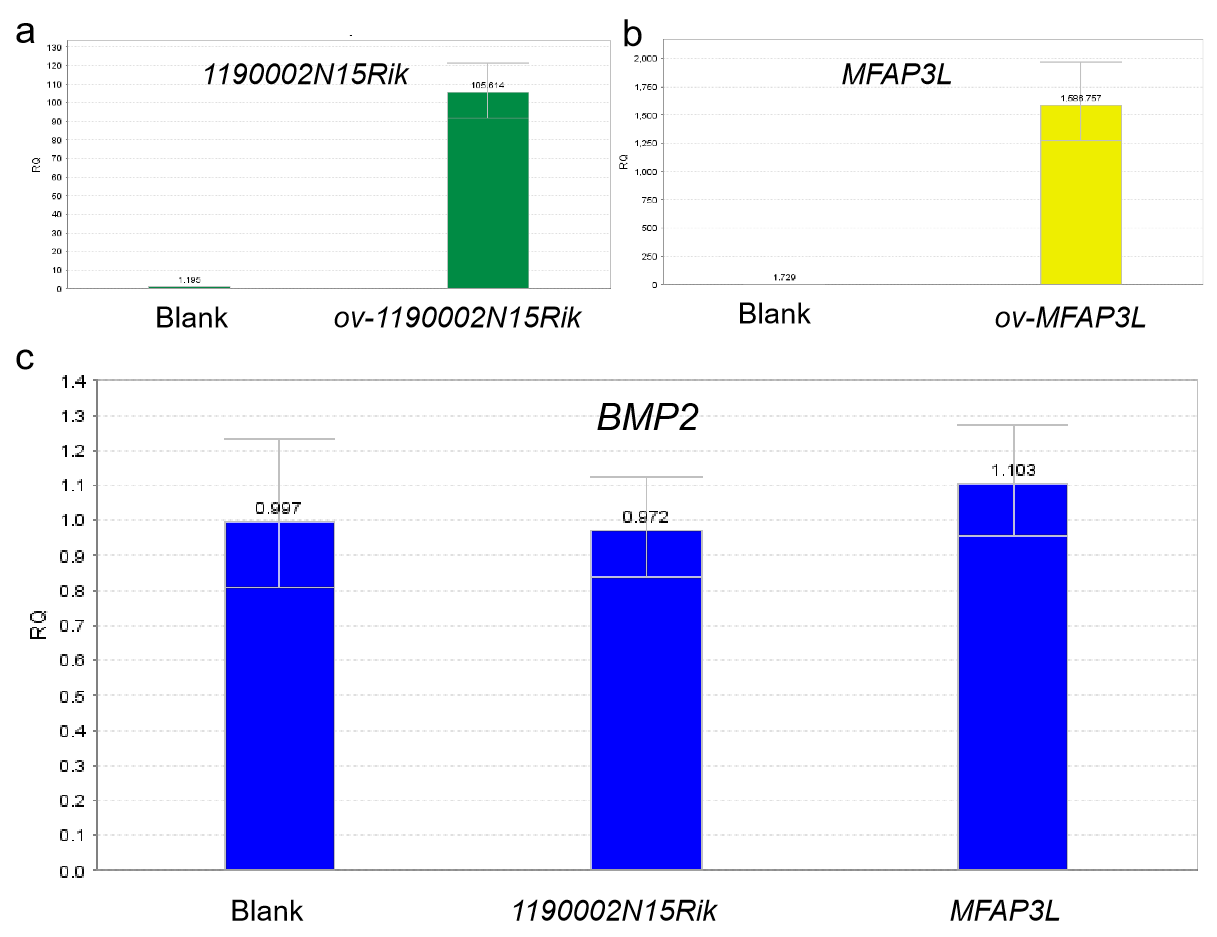


c

Fig S2 *BMP2* mRNA level changes after overexpressing *1190002N15Rik* and *MFAP3L* in C28/I2. a The over expression of *1190002N15Rik* was successfully achieved in C28/I2 cells*.* b The over expression of *MFAP3L* was successfully achieved in C28/I2 cells. c The expression levels of *BMP2* were examined in three cell groups: the control, overexpression of *1190002N15Rik* and overexpression of *MFAP3L*
